# Quantitation of α-Dicarbonyls, Lysine- and Arginine-Derived Advanced Glycation End Products, in Commercial Canned Meat and Seafood Products

**DOI:** 10.1021/acs.jafc.3c01205

**Published:** 2023-04-24

**Authors:** You-Yu Lin, Shih-Fang Huang, Kai-Wei Liao, Chi-Tang Ho, Wei-Lun Hung

**Affiliations:** † Master Program in Food Safety, College of Nutrition, Taipei Medical University, Taipei 11031, Taiwan; ‡ School of Food Safety, College of Nutrition, Taipei Medical University, Taipei 11031, Taiwan; § Department of Food Science, Rutgers University, New Brunswick, New Jersey 08901, United States

**Keywords:** advanced glycation end products, α-dicarbonyls, commercial canned food products, nutrients

## Abstract

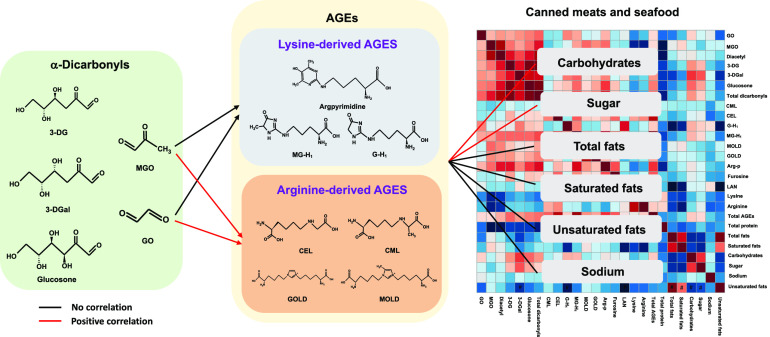

Commercial sterilization is a thermal processing method commonly used in low-acid canned food products. Meanwhile, heat treatment can significantly promote advanced glycation end product (AGE) formation in foodstuffs. In this research, the validated analytical methods have been developed to quantitate both lysine- and arginine-derived AGEs and their precursors, α-dicarbonyls, in various types of commercial canned meat and seafood products. Methylglyoxal-hydroimidazolone 1 was the most abundant AGEs found in the canned food products, followed by *N*ε-(carboxyethyl)lysine, *N*ε-(carboxymethyl)lysine, and glyoxal-hydroimidazolone 1. Correlation analysis revealed that methylglyoxal and glyoxal were only positively associated with the corresponding arginine-derived AGEs, while their correlations with the corresponding lysine-derived AGEs were not significant. Importantly, we demonstrated for the first time that total sugar and carbohydrate contents might serve as the potential markers for the prediction of total AGEs in canned meats and seafoods. Altogether, this study provided a more complete view of AGEs’ occurrence in commercial canned food products.

## Introduction

The Maillard reaction (MR) is a non-enzymatic browning reaction that provides the desirable color and flavor to thermally processed foods. However, research has shown that the potential harmful compounds may also be generated during this reaction such as reactive carbonyl species (RCS) and advanced glycation end products (AGEs). During MR, the reducing sugar reacts with the amine to give a Amadori compound, which further generates various types of RCS, such as 3-deoxyglucosone (3-DG), methylglyoxal (MGO), and glyoxal (GO).^1^ Previous studies have shown that food-derived RCS have a positive link with chronic diseases, such as certain types of cancer.^2^ Meanwhile, RCS formed in MR are the potent glycating agents that can further react with amino acids, particularly arginine or lysine, to form AGEs. *N*ε-(carboxymethyl)lysine (CML) is the first AGE to be identified in food and is formed from the reaction of GO and lysine.^3^ Similarly, *N*ε-(carboxyethyl)lysine (CEL) is a homologue of CML generated from the reaction of MGO and lysine. Along with CML and CEL, methylglyoxal lysine dimer (MOLD) and glyoxal lysine dimer (GOLD) are the cross-linked AGEs produced from reactions between two lysine side-chains and MGO and GO, respectively. In addition to lysine-derived AGEs, glycation of the arginine residue of protein is also a primary route to generate AGEs. Methylglyoxal-hydroimidazolone 1 (MG-H_1_) and glyoxal-hydroimidazolone 1 (G-H_1_) are the arginine-derived AGEs when arginine reacts with MGO and GO, respectively. Both lysine-derived and arginine-derived AGEs are ubiquitously present in foodstuffs, particularly thermally processed foods.

AGE was first isolated from a model system consisting of a sugar and an amino compound in 1980s.^4^ After being first described in relation to diabetic complications in 1990s,^5^ the implications of AGEs on human health and associated molecular mechanisms have been extensively studied. Along with AGEs formed during the glycation process in tissues endogenously, diet is a main source of exogenous AGEs, also known as the dietary AGEs (dAGEs). After fed with a high-AGEs baked chow diet, both free and protein-bound AGEs were profoundly accumulated in the plasma, kidney, and liver of mice, indicating that dAGEs could contribute to body’s AGEs pool.^6^ As a consequence, emerging data reveal that consumption of AGEs was positively correlated with several chronic diseases, particularly diabetes. In diabetic Goto-Kakizaki rats, oral administration of CML not only significantly increased fasting blood glucose, serum insulin, and oxidative stress but also disturbed several metabolic pathways including citric acid cycle, amino acid, and carbohydrate metabolism.^7^ Meanwhile, recent studies indicated that the unabsorbed dAGEs might be partially metabolized and degraded by gut microbiota. When SD rats were fed with a heat-treated AIN93 diet, the richness and diversity of gut microbiota significantly reduced.^8^ In their work, it is noteworthy that the abundance of *Ruminococcaceae* and *Alloprevotella*, which can produce short-chain fatty acids, significantly decreased due to the supplementation of dAGEs. Collectively, both absorbed AGEs accumulated in the tissue and unabsorbed AGEs present in the gastrointestinal tract could adversely impact human health.

Since dAGEs have emerged as a potential concern of human health, their occurrence in foodstuffs has become an important topic in the field of food science and nutrition. High contents of AGEs usually can be found in grains, confectionaries. and processed meats.^9^ Due to the fact that MR is a primary formation route of AGEs, processing temperature is a crucial factor that affects their contents in foodstuffs. A previous study indicated that heat treatment significantly increased the formation of CML in cooked beef, pork, chicken, salmon, and tilapia regardless of which processing method was used.^10^ Similarly, when fresh grass carp and catfish were heated in boiling water, protein-bound CML and CEL remarkably elevated along with the increased heating time, while the formation of free CML and CEL was not significantly affected.^11^ In addition to processing parameters, addition of sugars and salts and storage conditions might greatly affect AGEs formation in meat products.^12,13^ Interestingly, after pre-treatment with soy sauce, sour-sweet sauce, tomato sauce, and barbecue sauce, CML and pentosidine in cooked meats or seafoods profoundly increased.^14^

Commercial sterilization is a thermal processing method commonly used to achieve long-term shelf stability for canned foods. Due to the human health hazards of *Clostridium botulinum*, sterilization of foods in the low-acid category (pH > 4.6) is of primary concern. Low-acid and shelf-stable canned foods are usually commercially processed by thermal sterilization at temperatures around 121 °C to inactivate the heat-resistant spores of *C. botulinum*.^15^ Therefore, this severe thermal processing condition may cause AGEs formation in canned foods.^16^ In addition to AGEs, thermal processing may also promote the formation of furosine and lanthionine (LAN) because furosine is a marker of Amadori rearrangement products and LAN is a cross-linked amino acid derived from heat-induced protein modifications.^17^ Besides, various condiments (e.g., sauces, gravies, and food additives) are commonly added into canned meats and seafoods to enhance their flavor qualities and consumer acceptance, which may also greatly affect AGE formation.^12,18^ To better understand the contents of AGEs in canned meats and seafoods, a total of 11 AGEs in commercial canned meats and seafoods were quantitated in this study. Meanwhile, α-dicarbonyls, the primary precursors of AGEs, were also analyzed. Finally, correlation analysis among AGEs, nutrients (carbohydrates, sugars, proteins, and sodium), and MR products were also discussed to identify the potential indicators that can reflect AGE contents in canned food products.

## Methods and Materials

### Chemicals and Reagents

CML, CEL, MG-H_1_, G-H_1_, MOLD, GOLD, *N*(6)-{2-[(5-amino-5-carboxypentyl)amino]-2-oxoethyl}lysine (GOLA), glycolic acid lysine amide (GALA), pyrraline, argpyrimidine (Arg-p), pentosidine, d_4_-CML, and furosine were purchased form Iris Biotech (Marktredwitz, Germany). MGO (40% in aqueous solution), 2,3-hexanedione, arginine, lysine, d_4_-lysine, LAN, 2-methylquinoxaline, 2,3-dimethylquinoxaline, and furosine were purchased from Sigma (St. Louis, MO, USA). GO (40% in aqueous solution), diacetyl (DA), quinoxaline, and *o*-phenylenediamine (OPD) were purchased from Alfa Aesar (Great Britain, UK). 2-(1′,2′,3′,4′-Tetrahydroxybutyl)quinoxaline was purchased from Biosynth Carbosynth (St. Gallen, Switzerland). Glucosone, 3-deoxygalactosone (3-DGal), and 3-DG were purchased from Cayman (Ann Arbor, MI, USA). 2-(2′,3′,4′-trihydroxybutyl)quinoxaline was purchased from Toronto Research Chemicals (North York, Canada). Perchloric acid was purchased from Honeywell Fluka (Charlotte, NC, USA). All solvents used in this study are analytical or LC–MS grade and sourced from Sigma.

### Sample Preparation and AGE Extraction

A total of 64 commercial canned food products were purchased from a local supermarket (Taipei, Taiwan). A total of 10 groups of canned meats and seafood products was selected to investigate AGEs and α-dicarbonyls contents, including canned pork, chicken, spam, snail, saury, eel, mackerel, tuna, sardine, and clams. The sample was weighed after removal of the sauce. Two grams of canned foods were defatted with dichloromethane/*n*-hexane (4:1, v/v) prior to acid hydrolysis. The defatted sample was mixed with deionized water and then homogenized using a handheld homogenizer (IKA, Staufen, Germany). Then, 2 mL of 1 M sodium borohydride and 1 mL of 0.2 M sodium boric buffer were mixed with the sample. The mixture was allowed to stand at room temperature for 4 h to transform fructoselysine to hexitollysine. Thereafter, the mixture was mixed with 1.5 mL of 12 M HCl and hydrolyzed at 110 °C for 20 h. After cooling to room temperature, 100 μL of hydrolysate was spiked with 2 μL of d_4_-CML and 2 μL of d_4_-lysine and then dried using a centrifugal vacuum evaporator (SpeedVac, Thermo Scientific, Waltham, MA, USA). The dried sample was reconstituted with 200 μL water/acetonitrile (1:1) and filtered through a 0.22 μm nylon membrane prior to LC–MS analysis. 10 μL of the hydrolysate was diluted with 990 μL water/acetonitrile (1:1) prior to amino acid analysis.

### Determination of AGEs, Furosine, Amino Acids, and Lanthionine

Quantitation of AGEs, amino acids, furosine, and LAN in commercial canned meats and seafoods was carried out using a Waters UPLC system equipped with a Waters Xevo TQ-XS triple quadrupole electrospray ionization (ESI) tandem mass spectrometer (Milford, MA, USA). Chromatographic separations were performed using an Acquity BEH amide (2.1 × 100 mm, 1.7 μm, Waters) and a mobile phase consisting of 5 mM ammonium formate in 98% acetonitrile aqueous solution containing 0.1% formic acid (A) and 5 mM ammonium formate in 95% acetonitrile containing 0.1% formic acid (B). The column temperature was maintained at 60 °C. The flow rate and injection volume were set at 0.3 mL/min and 2 μL, respectively. The gradient program was set as follows: 0–15 min, 100–30% B, 15–20 min, 30% B, 15–16 min, 30–100% B, and 16–21 min, 100% B. Authentic standards were directly infused into the mass spectrometer and the product ions, cone voltage, and collision energy were optimized using IntelliStart software (Waters). The SRM transitions, cone voltage, collision energy, and retention time of AGEs, amino acids, and LAN are given in Table S1. The global MS parameters were set as follows: drying gas flow rate, 800 L/h; drying gas temperature, 600 °C; nebulizer gas pressure, 7 bar; and capillary voltage, 3900 V. Data analysis was performed using MassLynx software (Waters).

### Determination of α-Dicarbonyls

Extraction of α-dicarbonyls in canned foods were carried out as previously described, with slight modifications.^19^ Briefly, 30 μL of homogenate of canned food samples were mixed with 2 μL of 2,3-hexanedione as an internal standard and 120 μL of OPD (1 mg/mL 1.6 M perchloric acid) and then allowed to stand at room temperature for 20 h. After centrifugation at 12,000*g* for 20 min, the supernatant was collected and filtered through a 0.22 μm PVDF membrane prior to LC–MS analysis. To prepare the calibration curve of α-dicarbonyls, different concentrations of GO, MGO, DA, 3-DG, 3-DGal, and glucosone were mixed with OPD and then allowed to stand at room temperature for 20 h. Quantitation of α-dicarbonyls was performed using a Waters UPLC system equipped with a Waters Xevo TQ-XS triple quadrupole ESI tandem mass spectrometer. Chromatographic separations were performed using an Acquity BEH C18 column (2.1 × 100 mm, 1.7 μm, Waters) and 0.1% formic acid in water (a) and 0.1% formic acid in acetonitrile. The column temperature was maintained at 30 °C. The flow rate and injection volume were set at 0.2 mL/min and 2 μL, respectively. The gradient program was set as follows: 0–12 min, 2–15% B; 12–16 min, 15–100% B; and 16–18 min, 100% B. Authentic standards of derivatives of α-dicarbonyls, including quinoxaline, 2-methylquinoxaline, 2,3-dimethylquinoxaline, 2-(1′,2′,3′,4′-tetrahydroxybutyl)quinoxaline, and 2-(2′,3′,4′-trihydroxybutyl)quinoxaline, were directly infused into the mass spectrometer, and the product ions, cone voltage, and collision energy were optimized using IntelliStart software (Waters). The SRM transitions, cone voltages, collision energy, and retention time of α-dicarbonyls are given in Table S1. The global MS parameters were set as follows: drying gas flow rate, 800 L/h; drying gas temperature, 600 °C; nebulizer gas pressure, 7 bar; and capillary voltage, 3900 V. Data analysis was performed using MassLynx software (Waters).

### Method Validation

Linearity of AGEs, furosine, LAN, amino acids, and α-dicarbonyls was performed in water based on a calibration curve and their concentration ranges are given in Table 1. The LOD and LOQ were determined by injection of a series of dilute solutions of known levels of the analyte and the criteria of LOD and LOQ was set at 3 times and 10 times the signal to noise ratio, respectively. The reproducibility of the analytical method was confirmed by determination of intra- and inter-day precision and accuracy. The inter- and intra-day accuracy and precision were performed at three different concentrations of the analyte in the concentration range of a calibration curve and triplicate analyses were carried out on the same day or on 3 consecutive days. The recovery and matrix effect were evaluated by standard addition at low and high concentrations of the analyte into the canned chicken meat and analyzed in four replicates. For estimating matrix effect, the extract of the canned chicken meat was spiked with low and high concentrations of analytes and analyzed in four replicates. Recovery and matrix effect were calculated as described by our previous work.^20^

**Table 1:** Linearity, LOD, LOQ, Precision, and Accuracy of AGEs, LAN, Furosine, Amino Acids, and α-Dicarbonyls

						precision		accuracy	
compound	LOD (ng/mL)	LOQ (ng/mL)	calibration range (ng/mL)	linear equation	*r*^2^	intra-day (RSD %)	inter-day (RSD %)	intra-day (%)	inter-day (%)
AGEs									
CML	0.15	0.30	0.30–5000	2.48587*x* – 0.272377	0.999	3.2–5.1	4.1–9.2	92.3–106.1	90.2–101.4
CEL	0.15	0.30	0.30–5000	4.94461*x* – 0.163171	0.999	0.9–4.8	2.9–3.8	93.7–118.8	89.2–117.2
G-H_1_	0.30	0.61	0.61–2500	3.60658*x* – 1.07867	0.999	2.9–8.0	5.7–8.1	101.5–117.1	101.8–118.2
MG-H_1_	1.22	2.44	2.44–5000	3.02838*x* – 5.62019	0.999	0.8–6.9	6.9–7.3	96.0–114.0	96.0–118.5
MOLD	4.88	9.77	9.77–625	2.49537*x* – 7.79851	0.996	4.7–12.1	12.3–19.0	80.4–107.4	80.4–118.9
GOLD	2.44	4.88	4.88–625	2.98049*x* + 0.31141	0.991	0.9–12.2	5.2–15.9	82.7–107.0	85.0–118.4
Arg-p	0.04	0.15	2.44–2500	5.86432*x* – 5.9102	0.999	1.5–5.5	5.0–8.1	83.8–107.4	83.4–107.4
Others									
furosine	0.04	0.08	2.44–1250	17.7142*x* – 38.1347	0.990	4.6–10.1	3.9–15.3	85.9–103.5	84.2–108.1
LAN	0.61	1.22	1.22–5000	2.43151 + 2.36941	0.999	4.4–7.9	6.3–8.4	97.0–113.5	98.6–118.5
Amino acids									
lysine	4.88	9.77	19.53–5000	0.862362*x* + 16.9306	0.999	1.8–7.8	13.3–19.7	90.8–118.3	80.7–118.3
arginine	2.44	4.88	19.53–5000	4.10481*x* + 61.6338	0.993	1.3–6.9	10.1–14.5	83.2–120.0	82.0–119.6
α-Dicarbonyls									
GO	0.49	0.98	1.95–1000	0.00987321*x* + 0.00507433	0.999	4.9–11.4	3.3–4.4	87.0–110.9	91.9–108.7
MGO	0.98	1.95	1.95–500	0.0428563*x* + 0.174858	0.992	7.8–11.6	1.7–5.5	94.6–119.4	93.4–116.4
DA	0.49	0.98	1.95–250	0.0602038*x* + 0.178671	0.994	10.1–13.4	3.7–7.1	82.9–119.7	80.64–109.4
3-DG	0.06	0.12	0.24–1000	0.0712408*x* + 0.0061975	0.997	9.4–14.7	1.26–12.1	83.2–114.4	81.6–114.4
3-DGal	0.06	0.24	0.24–1000	0.0377808*x* + 0.000390898	0.994	9.0–13.7	2.6–8.5	84.5–110.4	80.5–110.4
glucosone	1.22	2.44	2.24–1000	0.0340243*x* – 0.00460714	0.999	2.7–5.9	6.4–10.5	82.9–107.3	82.1–107.3

### Statistical Analysis

The data are expressed as means ± standard deviations. Significant differences were statistically detected by one-way analysis of variance, followed by Tukey’s multiple comparison test (*p* < 0.05). Pearson’s correlation coefficients were performed using SPSS software (IBM, Armonk, USA) to assess the correlations between α-dicarbonyls, AGEs, and their nutrients.

## Results

### Method Development

In the present work, an UPLC system equipped a triple quadrupole ESI tandem mass spectrometer was used to determine a total of 11 AGEs, lysine, arginine, furosine (an indicator of Amadori product), and LAN (a cross-linked amino acid) in canned meats and seafoods. It should be noted that only seven AGEs were detected in canned meat and seafood products, including CML, CEL, MG-H_1_, G-H_1_, Arg-p, MOLD, and GOLD. The chromatograms of seven AGEs, lysine, arginine, LAN, and furosine are provided in Figure S1. Retention times of these analytes ranged from 5.77 to 11.20 min. The analytes separated well, and no significant interference was observed around retention times of the analytes. In the chromatogram of CML-d_4_, it should be noted that an additional peak was found. However, quantification of CML-d_4_ was not affected due to a clear separation from each other. Calibration curves of CML and CEL were prepared with different concentration levels ranging from 0.3 to 5000 ng/mL, while the concentration levels of MG-H_1_ and G-H_1_ ranged from 2.44 to 5000 ng/mL and 0.61 to 5000 ng/mL, respectively. For MOLD and GOLD, their concentration level prepared ranged from 4.88 to 625 ng/mL. Lysine and arginine are two primary precursors of AGEs and their levels were also quantitated in this study. The linearity of AGEs, furosine, LAN, lysine, and arginine exhibited good linearity (*r*^2^ > 0.990) over the concentration ranges. The LOD and LOQ of AGEs, furosine, LAN, and amino acids were 0.04–4.88 and 0.15–9.77 ng/mL, respectively. The intra-day and inter-day precision of AGEs, furosine, LAN, and amino acids were 0.80–12.2 and 2.9–19.7%, respectively. Meanwhile, their intra-day and inter-day accuracies were 80.4–120.0 and 80.4–119.6%, respectively. The mean recovery of AGE, furosine, LAN, and amino acids was 87.29–118.66%. The average matrix effect of all analytes was 93.13–116.52% (Table 2).

**Table 2:** Matrix Effect and Recovery of AGEs in Canned Chicken

	matrix effect		recovery	
compound	mean (%)	RSD (%)	mean (%)	RSD (%)
AGEs				
CML	113.79	0.40	106.73	7.42
CEL	93.13	2.30	89.45	11.68
G-H_1_	116.52	2.76	92.38	0.13
MG-H_1_	103.57	2.75	95.53	15.97
MOLD	95.60	0.64	96.98	4.21
GOLD	99.04	1.66	118.58	1.02
Arg-p	103.31	6.35	118.66	0.19
Others				
furosine	115.68	5.24	87.29	6.31
LAN	107.11	5.26	94.03	7.91
Amino acids				
lysine	101.21	2.71	105.32	2.80
arginine	105.61	1.00	101.17	15.17
α-Dicarbonyls				
GO	85.27	4.74	116.18	1.50
MGO	106.76	0.49	102.55	4.26
DA	102.94	3.12	84.83	4.41
3-DG	96.83	6.24	94.65	0.36
glucosone	93.50	6.74	98.29	5.57

Since α-dicarbonyls are the primary precursors of AGEs, a validated analytical method was also developed to quantitate their levels in canned foods. A total of six α-dicarbonyls were determined in the study, including GO, MGO, DA, 3-DG, 3-DGal, and glucosone. A derivatization reaction of α-dicarbonyls was required and their corresponding quinoxalines were able to be determined using LC–MS. The chromatograms of the derivatives of α-dicarbonyls are shown in Figure S2. It should be noted that the standard reference of derivatized 3-DGal is not commercially available; therefore, 3-DG and 3-DGal shared the same SRM parameters. All α-dicarbonyls exhibited good linearity (*r*^2^ > 0.992) over the concentration ranges (Table 1). The LOD and LOQ of α-dicarbonyls were 0.06–1.22 and 0.12–2.44 ng/mL, respectively. The intra-day and inter-day precision of α-dicarbonyls ranged from 2.68 to 14.66 and 1.26 to 12.06%, respectively. The intra-day and intra-day accuracies of α-dicarbonyls were 82.90–119.7 and 80.50–116.40%, respectively. The mean recovery of α-dicarbonyls ranged from 84.83 to 116.18% and their RSD was lower than 5.57%. The mean matrix effect of α-dicarbonyls ranged from 85.27 to 106.76%. It should be noted that the matrix effect of 3-DGal cannot be calculated because the quinoxaline derivative of 3-DGal is not commercially available.

### AGEs, Furosine, LAN, and Amino Acids in Canned Meats and Seafood

**Figure 1:**
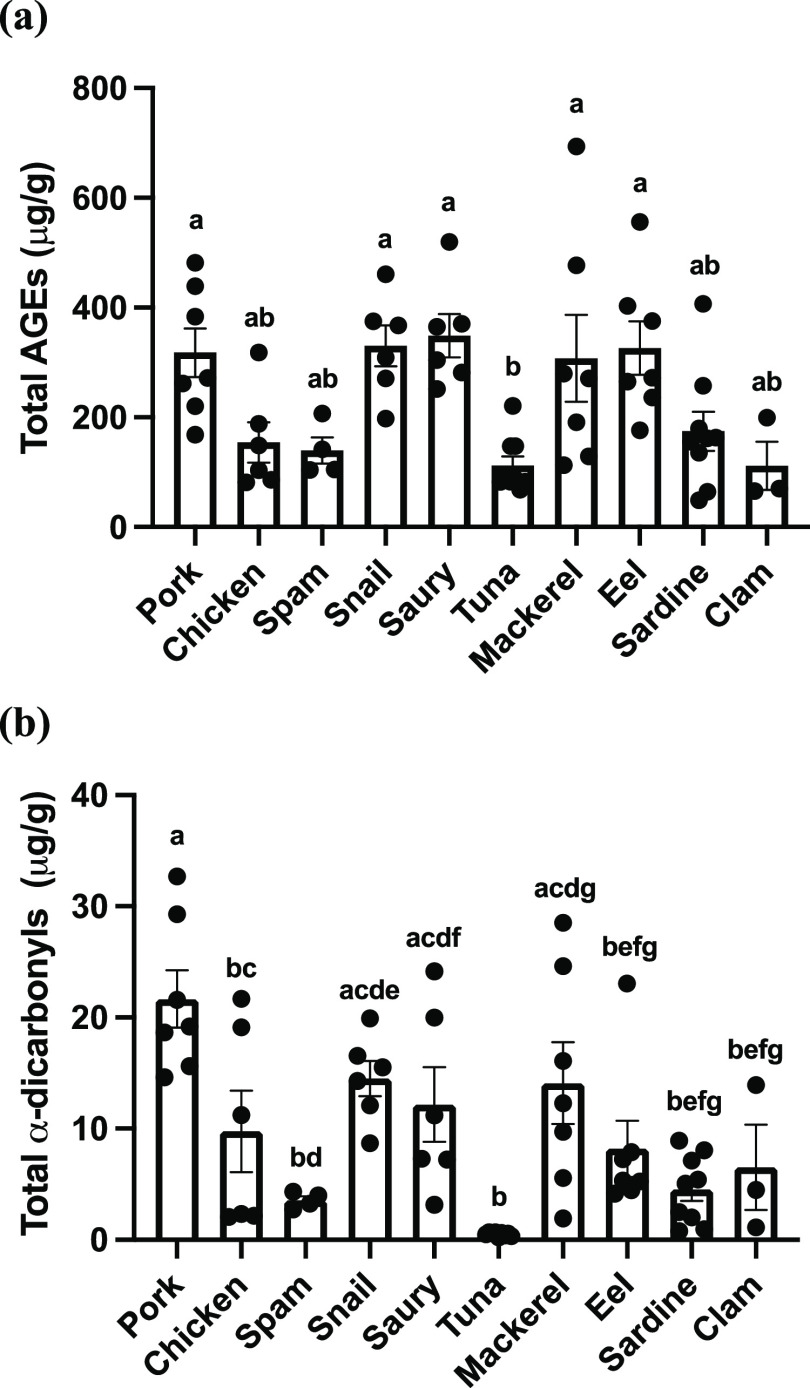
(a) Total AGEs and (b) total α-dicarbonyls in canned meat and seafood products. Data are expressed as means ± SDs. Different letters of the alphabet represent significant differences among different types of canned food products (*p* < 0.05).

The analytical method developed in this study was further applied to the screening of AGEs in canned foods. A total of 10 groups of canned meat and seafood products were analyzed. Among the AGEs detected in canned food products, CML, CEL, MG-H_1_, and G-H_1_ were found in all samples regardless of which groups of canned products. MG-H_1_ and CEL were the most abundant AGEs found in canned meat and seafood products. The highest levels of CEL and MG-H_1_ were found in canned mackerel and pork (278.51 and 323.94 μg/g, respectively), while the lowest levels of CEL and MG-H_1_ were found in the canned clam and sardine, respectively (11.76 μg CEL/g and 15.42 μg MG-H_1_/g). Along with CEL and MG-H_1_, CML was also ubiquitously present in all canned samples. The highest level of CML was found in the canned sardine (129.89 μg/g), while its lowest amount was found in the canned clam (4.25 μg/g). In the arginine-derived AGEs, we showed that the levels of G-H_1_ in canned foods were much lower than MG-H_1_. The highest amounts of G-H_1_ were found in the canned snail and clam (24.86 and 22.61 μg/g, respectively). Along with CML and CEL, MOLD and GOLD are also lysine-derived AGEs. However, MOLD and GOLD could not be found in some canned samples, particularly canned sardines, clams, and tuna. The highest levels of MOLD and GOLD were found in pork (15.77 and 33.09 μg/g). The levels of Arg-p in canned food products were relatively low and it could not be detected in all canned tuna, sardine, and clam samples. The total AGEs in canned foods were also calculated, and the results are shown in Figure 1. The highest amounts of total AGEs were observed in canned pork, snail, saury, mackerel, and eel (318.05, 330.32, 348.93, 307.69, and 326.40 μg/g for mean values, respectively), while the total AGEs in canned spam, chicken, tuna, and clam were relatively low (139.42, 154.50, 112.48, and 111.65 μg/g for mean values, respectively). The total AGEs in canned tuna were significantly lower than that from canned pork, snail, saury, mackerel, and eel (*p* < 0.05).

Lysine and arginine are the primary precursors of AGEs and their concentrations in canned food samples were also analyzed. The results showed that the highest levels of lysine and arginine were found in the canned chicken sample (848.49 and 331.07 μg/100 g, respectively), while the lowest levels of lysine and arginine were found in the canned clam and spam (89.78 and 49.09 μg/100 g, respectively). In addition, the indicators of Amadori products (furosine) and heat-induced protein modifications (LAN) were also analyzed. The highest levels of LAN were found in the canned snails and their levels ranged from 306.48–633.42 μg/g. The highest amount of furosine was found in the canned mackerel (103.16 μg/g) and its levels in canned tuna were relatively low (0.36–0.76 μg/g).

### α-Dicarbonyls in Canned Meats and Seafoods

A total of six α-dicarbonyls were analyzed in this study including MGO, GO, DA, 3-DG, 3-DGal, and glucosone. Among these α-dicarbonyls, MGO, DA, 3-DG, and 3-DGal were found in all canned food samples (Table 3). 3-DG was the most abundant α-dicarbonyl found in canned meat and seafood products. The highest amounts of 3-DG were found in canned pork, mackerel, and saury (18.98, 16.24, and 14.55 μg/g, respectively), while the lowest levels of 3-DG were found in canned tuna and sardine (0.03 μg/g). Similar to these results, the highest levels of MGO were found in canned pork, saury, and mackerel (10.73, 4.77, and 4.03 μg/g, respectively), while its lowest amounts were found in tuna, eel, and sardine (0.13, 0.38, and 0.48 μg/g, respectively). Meanwhile, the highest amounts of GO and 3-DGal were found in canned snail and mackerel (3.7 and 5.45 μg/g for snail and 2.56 and 2.38 μg/g for mackerel). It is noteworthy that GO was absent in all canned tuna samples. The highest levels of glucosone were found in canned mackerel and pork (3.47 and 3.26 μg/g, respectively), while glucosone was not found in some canned tuna, sardine, and clam samples. The results of total α-dicarbonyls are given in Figure 1. Canned pork, snail, and mackerel contained the highest amounts of total α-dicarbonyls (21.66, 14.51, and 14.10 μg/g, respectively), while the lowest level of total α-dicarbonyls was found in canned tuna (0.50 μg/g).

**Table 3:** AGEs, LAN, Furosine, Amino Acids, and α-Dicarbonyls in Canned Meats and Seafood^a^

μg/g	pork	chicken	spam	snail	saury	eel	mackerel	tuna	sardine	clam
sample number	7	6	4	6	6	7	7	9	9	3
AGEs										
CML	25.08–35.83	5.03–35.74	12.06–20.66	18.87–32.94	39.82–60.88	21.99–97.76	23.42–80.88	9.62–40.65	13.32–129.89	4.25–44.19
CEL	47.20–130.36	22.58–132.04	27.60–79.22	29.23–121.49	93.28–255.70	82.52–189.37	34.34–278.51	35.77–129.61	16.42–135.16	11.76–60.39
MG-H_1_	82.55–323.94	26.67–140.54	53.59–94.00	128.73–285.31	93.30–191.40	65.92–241.42	43.76–300.30	19.02–46.77	15.42–130.17	33.96–66.56
G-H_1_	4.68–12.94	3.07–7.38	3.91–8.93	14.49–24.86	6.13–11.32	2.27–7.41	5.58–14.52	2.76–4.02	1.06–7.64	6.78–22.61
MOLD	2.04–15.77	0.81–2.85	0.71–1.54	0.75–2.49	0.88–1.79	N.D–5.26	N.D–7.92	N.D	N.D–6.47	N.D–0.63
GOLD	2.24–33.09	1.03–2.28	0.99–2.88	0.55–4.29	2.61–4.88	1.30–16.54	0.49–22.09	N.D	N.D–11.91	N.D–5.18
Arg-p	0.27–0.87	N.D–0.22	N.D–0.06	0.07–0.55	N.D–0.26	ND–0.55	N.D–0.94	N.D	N.D	N.D
Others										
furosine	3.83–29.88	0.65–14.04	2.21–12.19	2.81–19.10	10.38–32.66	1.62–21.83	2.02–103.16	0.36–0.76	N.D–8.54	0.35–15.46
LAN	76.49–198.57	62.69–341.65	156.41–314.68	306.48–633.42	140.79–244.35	166.33–209.78	90.64–246.75	71.15–267.25	90.28–244.94	86.19–493.52
Amino acids										
lysine^a^	92.27–213.70	103.55–848.49	100.74–395.41	279.76–347.02	261.75–453.28	375.52–578.04	275.90–390.41	221.73–599.85	230.25–671.33	89.78–359.95
arginine^a^	51.69–80.75	63.77–331.07	49.09–144.05	170.72–208.08	193.92–277.26	182.25–256.26	189.16–290.77	136.12–261.43	147.36–293.49	89.48–174.75
α-Dicarbonyls										
GO	0.82–2.05	0.18–1.55	0.19–0.66	1.40–3.70	0.13–1.03	0.13–1.54	0.21–5.45	N.D	0.05–0.56	0.15–1.87
MGO	4.10–10.73	1.43–3.99	1.52–2.75	1.78–3.53	0.76–4.77	0.38–1.85	0.80–4.03	0.13–0.49	0.48–4.73	0.78–3.12
DA	0.65–2.01	0.17–1.47	0.16–0.28	0.30–0.81	0.10–0.89	0.10–0.79	0.17–0.84	0.03–0.06	0.09–0.33	0.12–0.85
3-DG	5.82–18.98	0.05–11.79	0.21–1.11	0.20–9.57	1.54–14.55	2.21–13.92	0.34–16.24	0.03–0.21	0.03–5.72	0.06–5.43
3-DGal	0.68–1.36	0.03–1.65	0.08–0.30	0.17–2.56	0.11–0.63	0.65–2.06	0.04–2.38	0.02–0.03	0.02–0.52	0.02–1.30
glucosone	1.32–3.26	0.03–2.77	0.15–0.27	1.02–1.89	0.51–2.55	0.42–2.87	0.33–3.47	N.D–0.09	N.D–1.28	N.D–1.90

a Lysine and arginine concentrations are given as μg/100 g. N.D denotes not detected.

### Association between AGEs, α-Dicarbonyls, and Nutrients

**Figure 2:**
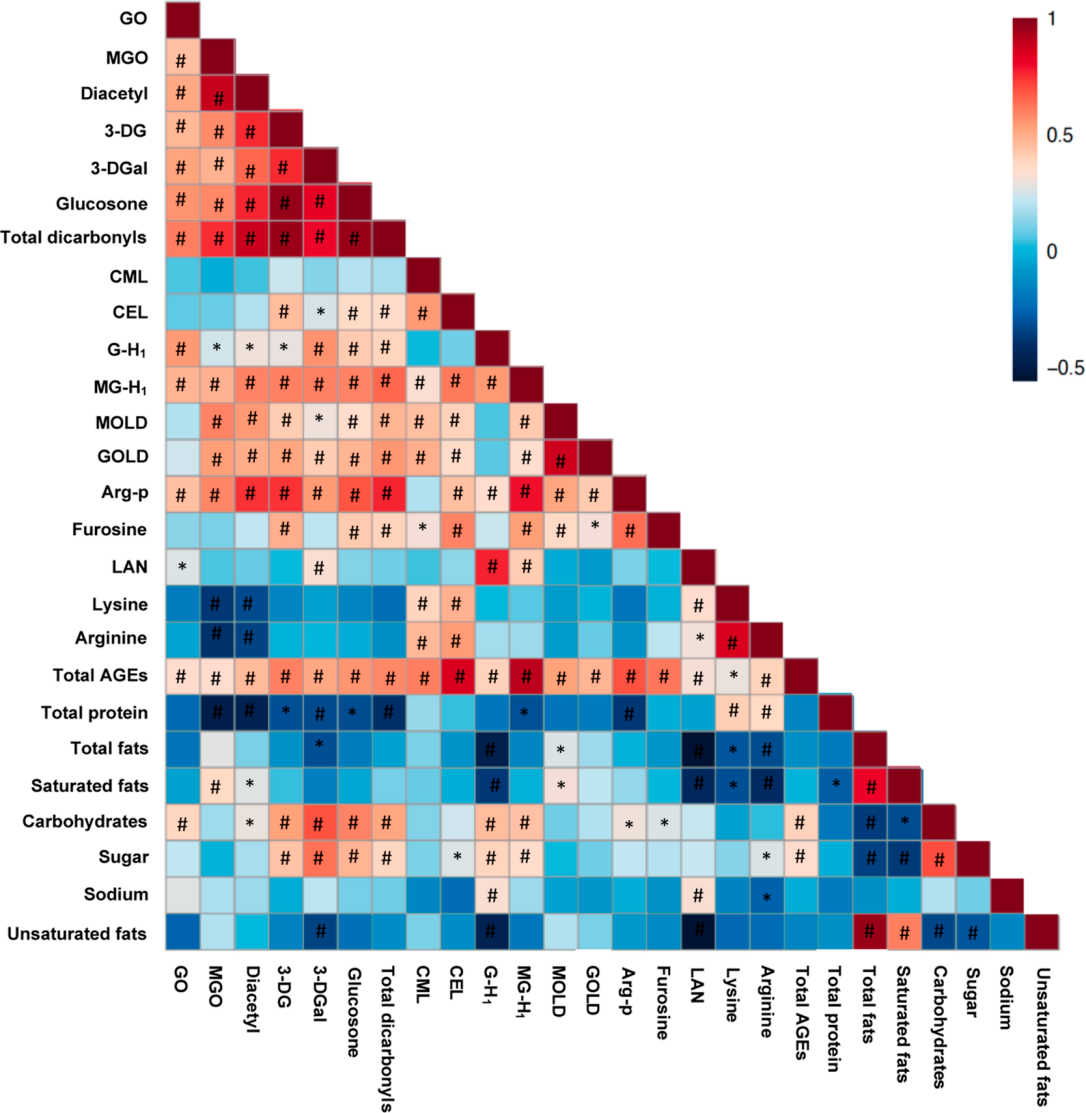
Heatmap of Pearson’s correlations among AGEs, α-dicarbonyls, and nutrients. *p < 0.05, ^#^*p* < 0.01.

**Figure 3:**
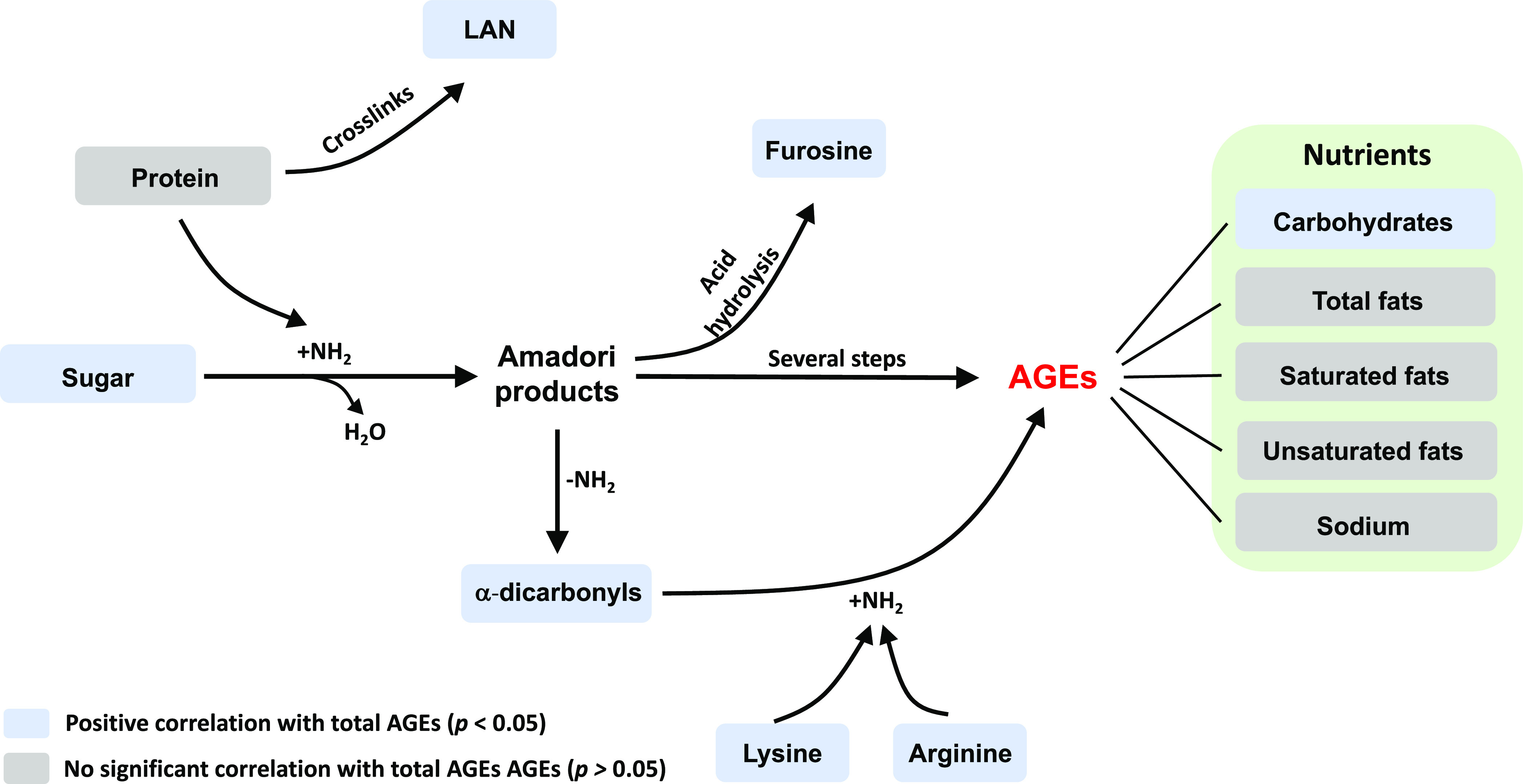
Correlations of AGEs with nutrients, LAN, furosine, and amino acids.

**Figure 4:**
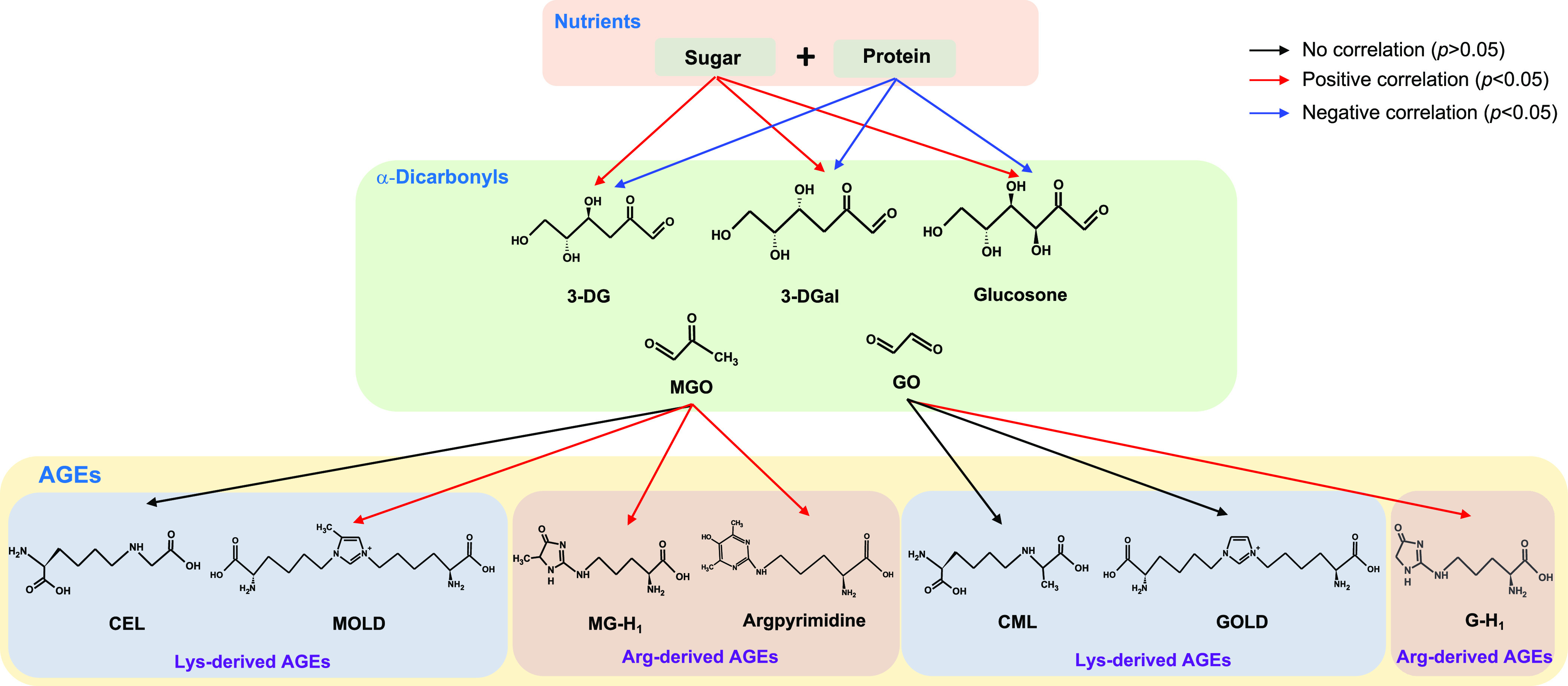
Correlations of α-dicarbonyls with sugar, protein, and AGEs.

The nutrition information (total protein, total fats, unsaturated fats, saturated fats, total carbohydrates, total sugar, and sodium) of canned meats and seafood are given in Table S2. To identify the correlations among AGEs, α-dicarbonyls, and nutrients, Pearson’s correlations were calculated and a corresponding heatmap was constructed based on the *p*-value matrix (Figure 2). Our results revealed that total AGEs of canned meats and seafood were positively associated with their total contents of carbohydrates and sugar (*p* < 0.05), while total AGEs were not significantly associated with total contents of protein, fats, unsaturated fats, saturated fats, and sodium. Meanwhile, total AGEs were also positively correlated with total contents of α-dicarbonyls, LAN, furosine, arginine, and lysine (*p* < 0.05). The formation pathways of AGEs and its correlations with nutrients, total RCS, amino acids, LAN, and furosine are shown in Figure 3. In addition, our results indicated that α-dicarbonyls, including 3-DG, glucosone, and 3-DGal, in canned meats and seafood were positively associated with their total contents of sugar, while these α-dicarbonyls were negatively correlated with total contents of protein (Figures 2 and 4). In this study, both MGO-derived and GO-derived AGEs were found in canned meats and seafoods; therefore, their correlations with MGO and GO were also discussed. Arginine-derived AGEs, including MG-H_1_, Arg-p, and G-H_1_, were positively associated with MGO and GO, respectively. In lysine-derived AGEs, only MOLD had a positive correlation with MGO, while CEL, CML, and GOLD were not significantly correlated with MGO and GO. Correlations between nutrients (sugar and protein)—α-dicarbonyls and α-dicarbonyls—lysine-derived and arginine-derived AGEs are summarized in Figure 4.

## Discussion

In this study, a validated analytical method has been successfully developed, in which a total of 11 AGEs were simultaneously quantified. Since AGEs are a unique group of highly polar compounds, they cannot be fully separated from each other when a reversed-phase column is used (e.g., C18) for analysis. For instance, both CML and CEL were not retained well in a reverse-phase column, in which their peaks were usually overlapped.^21,22^ To solve this problem, ion-pairing chromatographic methods have been used to enhance their separation in the column.^17^ However, long-term use of ion pairing reagents may degrade the performance of the ESI interface and suppress signal intensity of analytes.^23^ To avoid these issues, we used hydrophilic interaction liquid chromatography (HILIC) to separate AGEs, amino acids, LAN, and furosine. In HILIC, only formic acid and ammonium formate were added into the mobile phase instead of using surfactants as ion-pairing agents. We found that HILIC provided satisfactory chromatographic separations of most of analytes (Figure S1). Unlike AGEs, satisfactory separations of α-dicarbonyls can be achieved using a reversed-phase C18 column (Figure S2). AGEs, amino acid, furosine, LAN, and α-dicarbonyls exhibited good linearity (*r*^2^ > 0.99), intra- and inter-day precision, and accuracy. The LOD and LOQ of AGEs ranged from 0.04 to 4.88 and from 0.08 to 9.77 ng/mL, respectively. The mean matrix effect and recovery of AGEs ranged from 93.13 to 113.79 and from 89.45 to 118.66%, respectively. Similar to our current findings, a previous study used liquid chromatography coupled with quadrupole-Orbitrap mass spectrometry to analyze dietary AGEs in different foodstuffs and their results showed that the RSD (%) of inter- and intra-day precision ranged from 1.5 to 13.2 and from 2.5 to 30.7%, respectively.^17^ Also, the results also found that the LOQ of AGEs ranged from 2.04 to 29.19 ng/mL. Collectively, the analytical method developed in this study was suitable for quantitation of AGEs in canned meats and seafoods.

In MR, both MG-H_1_ and G-H_1_ are arginine-derived AGEs generated when lysine reacts with MGO and GO, respectively. However, information with respect to formation of arginine-derived AGEs in canned food products are still limited. To our knowledge, this is the first time that arginine-derived AGEs have been quantitated in various types of canned meats and seafoods. In this study, MG-H_1_ was the most abundant dAGEs in canned meats and seafoods, while the levels of G-H_1_ were relatively low (Table 3). In animal-derived foodstuffs, previous studies have shown that MG-H_1_, an arginine-derived AGE, was the most abundant AGE along with lysine-derived AGEs. After deep-frying for 6 min, the primary AGE formed in minced-meat hot dog was MG-H_1_ (>30 mg/100 g), which was significantly higher than CEL and CML (<10 mg/100 g). In addition to animal-derived foodstuffs, MG-H_1_ is also a primary AGE found in plant-derived foodstuffs, such as peanuts, bread, cornflakes, and tea.^24−26^ It is noteworthy that the average intake of MG-H_1_ was 21.7 mg/day, which was remarkably higher than that of CML and CEL (3.1 and 2.3 mg/day, respectively).^27^

CML and CEL are the most abundant lysine-derived dAGEs ubiquitously present in foodstuffs. In this work, CML and CEL were found in all canned meat and seafood products. Consistent with our current findings, CML and CEL were also previously found in animal food products including, pork, beef, chicken, salmon, duck, tilapia, grass carp, catfish, sardines, mackerel, and milk.^10,11,28,29^ Meanwhile, in the partial least squares discriminant analysis model, no clear separations were found among the clusters of different types of canned food products (Figure S3). These results suggested that types of canned food were not a dominant factor that affects AGEs. Given that dAGEs are primarily generated from MR, research has shown that the thermal processing condition is a predominant factor that dominates AGE formation. A previous study indicated that the formation of CML and CEL in ground beef and minced hot dog was positively associated with heating time and temperature.^25,30^ Similarly, levels of CML, CEL, and MG-H_1_ in fish cakes increased with increased heating time regardless of which thermal processing method was used.^31^ Along with CML and CEL as lysine-derived AGEs, MOLD and GOLD are the cross-linked AGEs produced when MGO or GO react with two molecules of lysine. MOLD and GOLD were detected in most of canned meats and seafood and their levels were much lower than CML and CEL. Consistent with our findings, a recent study comprehensively investigated the formation of MGO- and GO-derived AGEs in meats and the results showed that the levels of CML and CEL in grilled porcine meats were also much higher than MOLD and GOLD.^32^ In addition, MOLD and GOLD were also found in chicken breast, beef sticks, and bakery products.^19,33,34^

In this study, CML, CEL, MG-H_1_, and G-H_1_ were found in all canned meats and seafoods. Recently, Yu et al. (2022) comprehensively analyzed the levels of CML and CEL in a total of 49 commercial meat products. The levels of CEL in canned pork found in their work ranged from 60.1 to 81.7 μg/g, which were similar to our results (47.2–130.36 μg/g). Also, Zhao et al. (2021) analyzed the concentrations of CML and CEL in different types of commercial canned fish.^35^ Their study revealed that the levels of CML in canned eel, saury, and tuna were 7.49, 4.52, and 3.82 μg/g, respectively.^35^ However, the lowest concentrations of CML in canned eel, saury, and tuna found in our current study were 21.99, 39.82 and 9.62 μg/g, respectively, which were higher than that from their findings. Also, high variations of CML, CEL, and MG-H_1_ were found in the same type of canned meats or seafoods, which results in a wide range of total AGEs (Figure 1). Except heating time and heating temperature as the crucial factors affecting AGE formation mentioned above, large variance of AGEs found in the same type of canned food products might be due to the addition of condiments, spice, and food additives. Sun et al. (2021) has reported that the addition of glucose, fructose, and lactose profoundly increased the formation of CML and CEL in ground pork after commercial sterilization. However, the levels of CML and CEL were not significantly affected by the addition of sucrose.^12^ Addition of reducing sugar profoundly promoted AGE formation might be due to the MR reaction, which is a primary formation route of AGEs. Therefore, low contents of AGEs and α-dicarbonyls found in canned tuna were possibly associated with their low sugar contents (Table S2). Correlation analysis of our work also revealed that total sugar contents of canned meats and seafoods were positively correlated with their total contents of AGEs (Figure 3). In addition to sugar, types of sodium salts have different impacts on accumulation of AGEs in meats. Niu et al. (2018) showed that the formation of CML in ground pork remarkably increased by the treatment with 1.5–5% NaCl, while the addition of NaNO_2_ reduced the formation of CML and CEL.^13^ Their results might explain why the correlation between total AGEs in canned meats and seafoods and their contents of sodium was not significant (Figure 3). Besides sugar and salts, decreased levels of CML and CEL were also observed in ground pork by the addition of citric acid and acetic acid.^18^ Spices commonly used in European cuisine effectively inhibited AGE formation in a bovine serum albumin–MGO assay.^36^ Since canned food products usually have a longer shelf life as compared to fresh food products, it is important to note that longer storage periods might also promote AGE formation. The levels of CML and CEL in vacuum-packaged Chinese sausages profoundly elevated after storage for 180 days.^37^ It is also interesting to note that CML, CEL, pyrraline, and GOLD in conventional and ultrahigh temperature-sterilized milk significantly accumulated after 1 year of storage.^29^ Altogether, processing conditions (e.g., heating time and temperature), addition of condiments (e.g., sugar), and storage periods are the important factors that might cause substantial impacts on AGEs formation in canned meats and seafood.

Due to the fact that the quantitation of AGEs in foodstuffs is time-consuming (e.g., acid hydrolysis) and expensive (e.g., LC–MS/MS), this study attempts to identify the potential indicators, particularly information provided from the nutrition facts label, which can reflect the total AGEs contents in canned meats and seafoods. From the nutrition facts label, we observe that only total contents of sugar and carbohydrates in canned meats and seafoods were positively correlated with their total AGEs (*p* < 0.05), while total contents of protein, sodium, fats, saturated fats, and unsaturated fats were not significantly correlated with total AGEs (Figure 3). These results suggest that AGE contents in canned meat and seafood products could be predicted by their total contents of sugar and carbohydrates shown on the nutrition facts label. In order to find the potential indicators, LAN and furosine were also quantitated in this study and the results showed that LAN and furosine were positively correlated with total AGEs (Figure 3). Furosine is widely used as a marker of Amadori rearrangement products and LAN is a cross-linked amino acid that commonly serves as an indicator of heat-induced protein modifications.^17^ Collectively, this work demonstrates for the first time that LAN and furosine might serve as the potential indicators of total AGE contents in canned meats and seafood.

Because α-dicarbonyls are the MR intermediates, their correlations with protein, sugar, and AGEs were also addressed in this study. 3-DG, glucosone, and 3-DGal were correlated with total sugar contents (Figure 4). Due to the fact that meats and seafoods are animal-derived foods which usually contain low contents of saccharides in comparison to plant-derived foods. Therefore, high contents of sugar found in canned meats and seafoods are possibly due to the addition of sugar-rich condiments. Sugar-rich condiments usually contained high concentrations of α-dicarbonyls, such as honey, sugar syrup, and apple molasses.^19^ Interestingly, the level of α-dicarbonyls in the sugar-free spiced cake was 95 mg/kg, which was much lower than that from the regular spiced cake of the same brand (543 mg/kg).^19^ Along with the condiment, thermal treatment can also cause accumulation of α-dicarbonyls in meats. A recent study indicates that the levels of MGO significantly accumulated in pork meat during grilling at 200 °C, while the level of GO decreased with increased heating time.^32^ Meanwhile, the levels of MGO in sausages were 7.92 and 3.55 μg/g after thermal treatment for 1 and 2 h, respectively.^38^ These results suggest that the formation of α-dicarbonyls may decrease during heating because they are the intermediates of MR. α-Dicarbonyls may be degraded under severe thermal conditions (e.g., roasting, baking, and deep frying). Similarly, thermal processing might cause protein degradation in both animal- and plant-derived food products. For instance, both deep-frying and air-drying led a significant loss of lysine in chicken breast.^39^ Also, the levels of amino acids in hazelnut significantly decreased after roasting at 170 °C.^40^ Notably, our work indicated that 3-DG, glucosone, and 3-DGal had a negative correlation with total protein contents (Figure 4). Importantly, although MGO and GO are the precursors of both lysine-derived and arginine-derived AGEs, only arginine-derived AGEs had positive correlations with MGO and GO. In lysine-derived AGEs, only a positive correlation was found between MOLD and MGO, while CEL, CML, and GOLD did not have significant correlations with their corresponding α-dicarbonyls (Figure 4). The possible formation pathways of CML include the Namiki pathway and the Hodge pathway.^29^ In the Namiki pathway, GO formed from degradation of Schiff bases reacts with the nucleophilic sites on proteins to generate CML. Alternatively, CML can also be formed from autooxidation of Amadori products without GO participation in the Hodge pathway. Therefore, lack of a significant correlation between CML and GO might be probably due to the Hodge pathway involved in CML formation in canned food products. However, more research is required to investigate the detailed formation mechanisms of lysine-derived AGEs and their correlations with MR products in canned food products.

In summary, this study gave a more comprehensive view with respect to the occurrence of AGEs in various types of canned meats and seafoods. To our knowledge, this is the first time that arginine-derived AGEs have been quantitated in canned meats and seafoods. Correlation analysis revealed that MGO and GO were significantly correlated with arginine-derived AGEs, while their correlations with lysine-derived AGEs were not significant. Importantly, we show that total contents of AGEs in canned meats and seafood were positively associated with their total contents of sugar and carbohydrates. Therefore, our current findings suggest that sugar and carbohydrate contents shown on the nutrition facts label might serve as potential indicators that can easily predict total AGE contents in commercial canned food products, particularly canned meats and seafoods.

## Supporting Information Available

Mass spectrometric settings and chromatograms of AGEs, lysine, arginine, LAN, furosine, and α-dicarbonyls; PLS-DA of AGE contents in different types of canned meats and seafood; and nutrient contents of canned meats and seafoods (PDF)

## Notes

Chemical compounds studied in this article are as follows: methylglyoxal (PubChem CID: 880), glyoxal (PubChem CID: 7860), 3-deoxyglucosone (PubChem CID: 114839), *N*ε-(carboxymethyl)lysine (PubChem CID: 123800), *N*ε-(carboxyethyl)lysine (PubChem CID: 23400779), methylglyoxal-hydroimidazolone 1 (PubChem CID: 135625302), glyoxal-hydroimidazolone 1 (PubChem CID: 101942492), lanthionine (PubChem CID: 98504), and furosine (PubChem CID: 123889).

## List of Abbreviations

3-DG: 3-deoxyglucosone
3-DGal: 3-deoxygalactosone
AGEs: advanced glycation end products
Arg-p: argpyrimidine
CEL: *N*ε-(carboxyethyl)lysine
CML: *N*ε-(carboxymethyl)lysine
DA: diacetyl
GALA: glycolic acid lysine amide
G-H_1_: glyoxal-hydroimidazolone 1
GO: glyoxal
GOLA: *N*(6)-[2-[(5-amino-5-carboxypentyl)amino]-2-oxoethyl]lysine
GOLD: glyoxal lysine dimer
MG-H_1_: methylglyoxal-hydroimidazolone 1
MGO: methylglyoxal
MOLD: methylglyoxal lysine dimer
MR: Maillard reaction
