# Supplementary material for: Quantitation of α-Dicarbonyls, Lysine- and Arginine-Derived Advanced Glycation End Products, in Commercial Canned Meat and Seafood Products
Source: J Agric Food Chem. 2023 Apr 24;71(17):6727–37. doi: 10.1021/acs.jafc.3c01205 (PMC10161224; doi:10.1021/acs.jafc.3c01205)
Supplement: Supplementary file 1 — jf3c01205_si_001.pdf [file jf3c01205_si_001.pdf]

Quantitation of α-dicarbonyls, lysine-derived and arginine-derived advanced glycation end products in commercial canned meat and seafood products

You-Yu Lin[a], Shih-Fang Huang[a], Kai-Wei Liao[b], Chi-Tang Ho[c], Wei-Lun Hung[b,*]

[a]Master Program in Food Safety, College of Nutrition, Taipei Medical University, Taipei 11031, Taiwan

[b]School of Food Safety, College of Nutrition, Taipei Medical University, Taipei 11031, Taiwan

[c]Department of Food Science, Rutgers University, New Brunswick, NJ, 08901, USA

# Supplementary Information

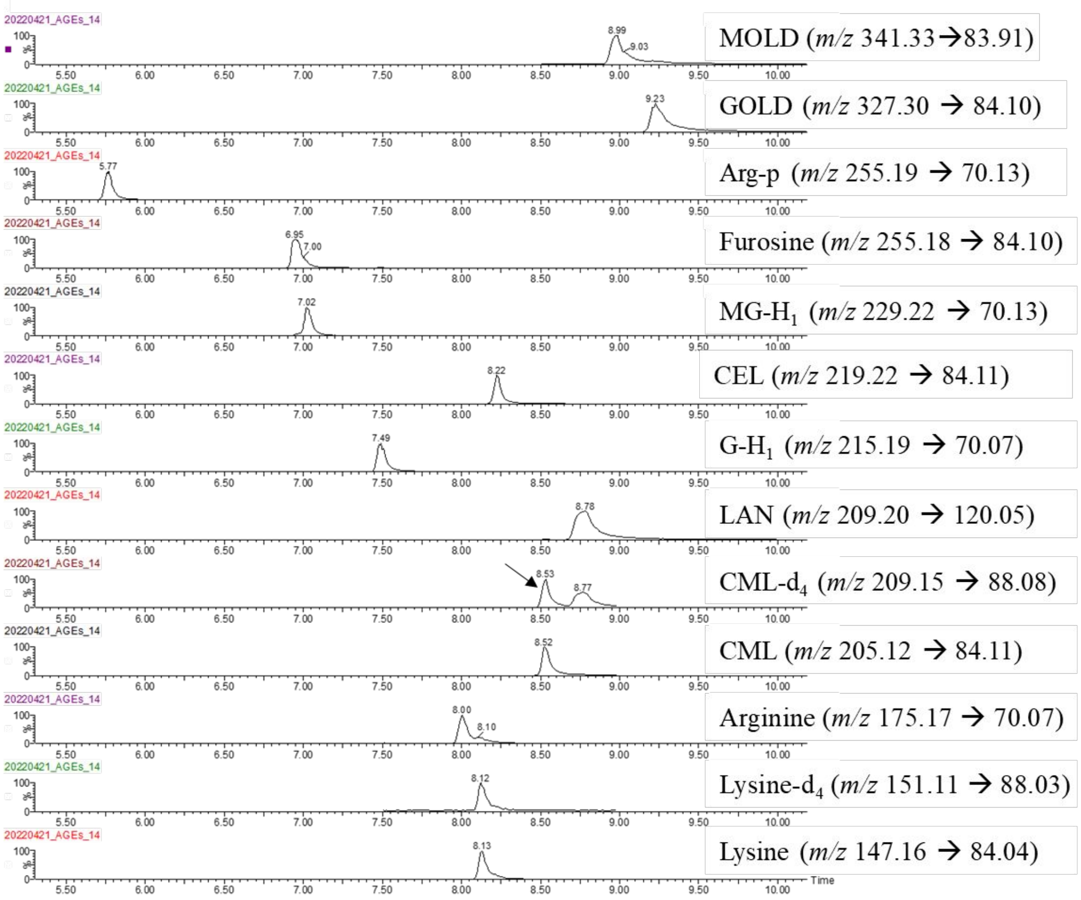


Figure S1. Chromatograms of AGEs, lysine, arginine, LAN and furosine

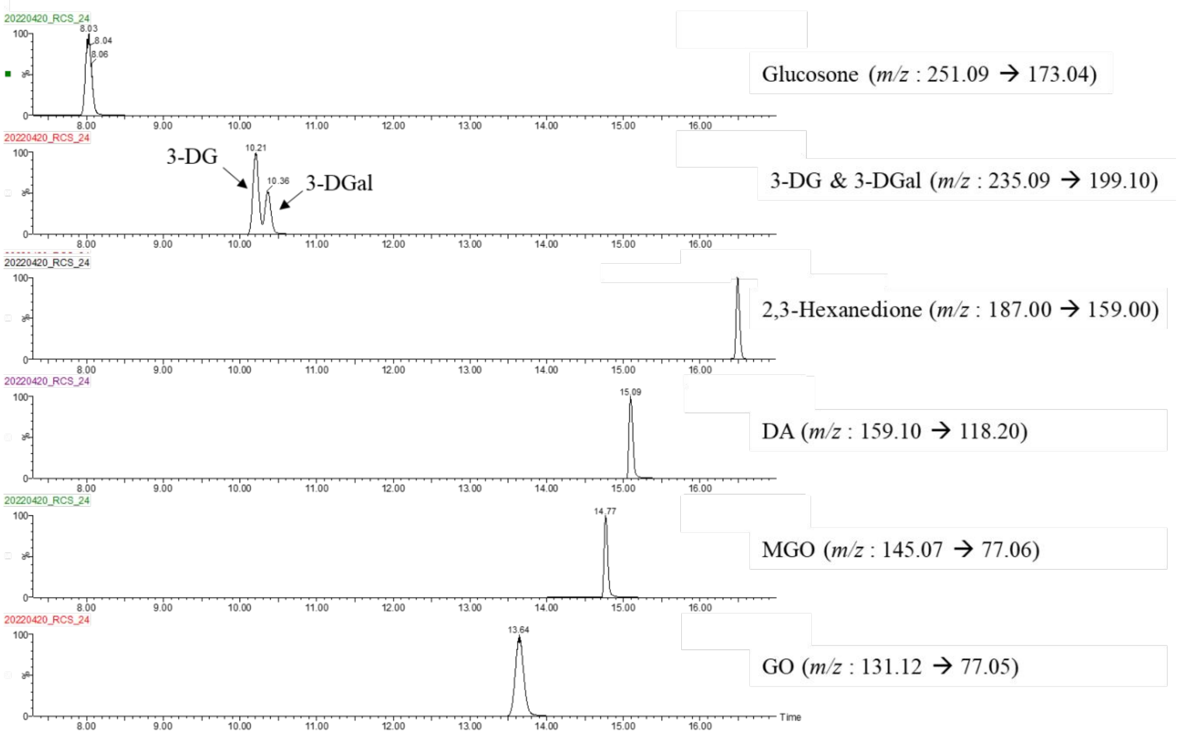


Figure S2. Chromatograms of α-dicarbonyls

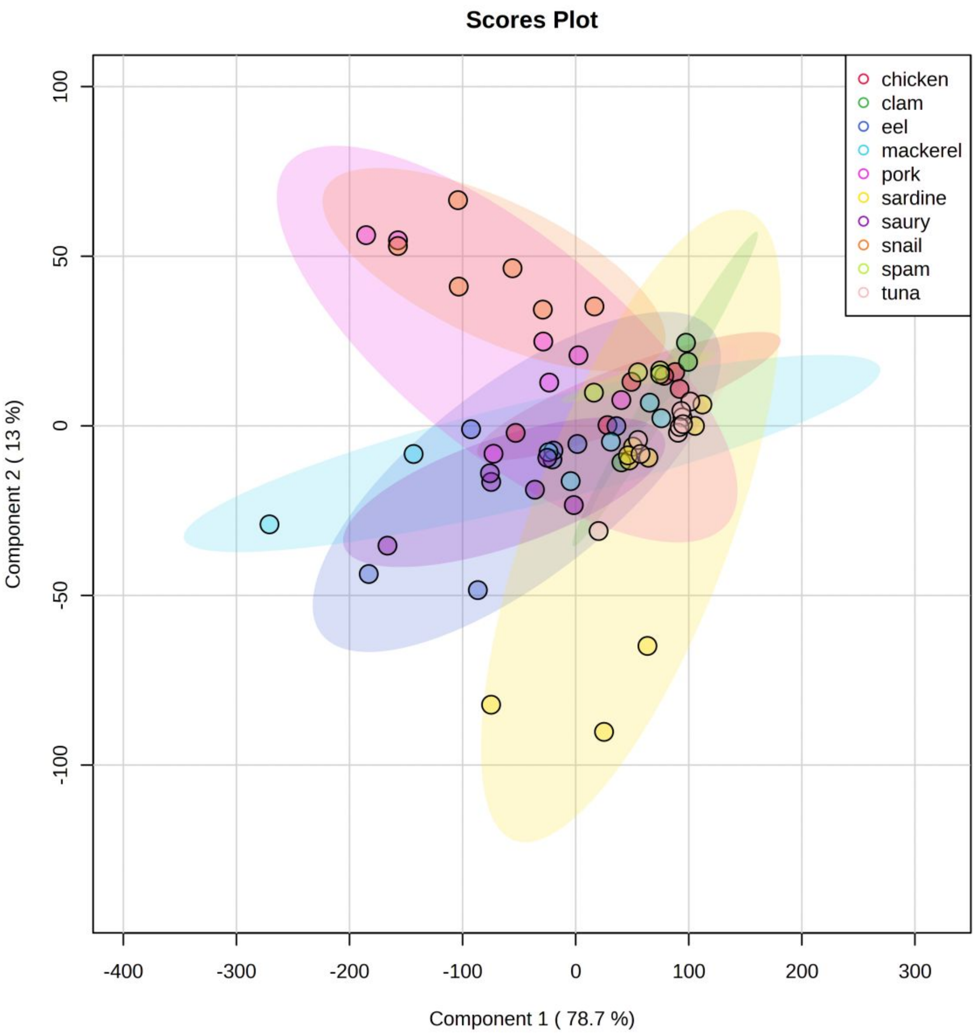


Figure S3. PLS-DA of AGEs contents in different types of canned meats and seafood

Table S1. Mass spectrometric settings for the standards and internal standards

| Compound | Precursor ion (m/z) | Product ion (m/z) | Cone (V) | Collision (V) | Retention Time (min) |
|---|---|---|---|---|---|
| ***AGEs*** | | | | | |
| CML | 205.12 | 84.11 | 4 | 24 | 8.52 |
| CEL | 219.22 | 84.11 | 26 | 22 | 8.22 |
| G-$H_1$ | 215.19 | 70.07 | 30 | 22 | 7.49 |
| MG-$H_1$ | 229.22 | 70.13 | 20 | 26 | 7.02 |
| MOLD | 341.33 | 83.91 | 68 | 42 | 8.99 |
| GOLD | 327.30 | 84.10 | 80 | 34 | 9.23 |
| Arg-p | 255.19 | 70.13 | 10 | 28 | 5.77 |
| GOLA | 333.30 | 84.10 | 16 | 36 | 11.20 |
| GALA | 205.19 | 142.09 | 14 | 16 | 7.38 |
| Pyrraline | 255.18 | 148.10 | 2 | 22 | 5.98 |
| Pentosidine | 379.30 | 187.09 | 24 | 42 | 10.83 |
| CML-$d_4$ | 209.15 | 88.08 | 30 | 24 | 8.53 |
| ***Amino acids*** | | | | | |
| Lysine | 147.16 | 84.04 | 32 | 18 | 8.13 |
| Arginine | 175.17 | 70.07 | 46 | 22 | 8.00 |
| Lysine-$d_4$ | 151.11 | 88.03 | 2 | 18 | 8.12 |
| ***Others*** | | | | | |
| Furosine | 255.18 | 84.10 | 8 | 26 | 6.95 |
| Lanthionine | 209.20 | 120.05 | 36 | 16 | 8.78 |
| ***α-dicarbonyls*** | | | | | |
| Glyoxal | 131.12 | 77.05 | 72 | 22 | 13.64 |
| Methylglyoxal | 145.07 | 77.06 | 6 | 24 | 14.77 |
| Diacetyl | 159.10 | 118.20 | 2 | 22 | 15.09 |
| 3-Deoxyglucosone | 235.09 | 199.10 | 18 | 18 | 10.21 |
| 3-Deoxygalactosone | 235.09 | 199.10 | 18 | 18 | 10.36 |
| Glucosone | 251.09 | 173.04 | 2 | 18 | 8.03 |
| 2,3-Hexanedione | 187.00 | 159.00 | 6 | 24 | 16.49 |

Table S2. Nutrient contents of canned meats and seafoods

| Type | no | Protein (g/100g) | Fat (g/100g) | Saturated fat (g/100g) | Unsaturated fat (g/100g) | Carbohydrate (g/100g) | Sugar (g/100g) | Sodium (mg/100g) | Processing |
|---|---|---|---|---|---|---|---|---|---|
| Pork | 1 | 11 | 32.1 | 11.9 | 20.2 | 5.9 | 0.7 | 414 | Sauteing |
| | 2 | 8.1 | 15.3 | 4.8 | 10.5 | 9.1 | 1 | 207 | Sauteing |
| | 3 | 13.3 | 23.3 | 9.5 | 13.8 | 7.1 | 3.4 | 486 | Sauteing |
| | 4 | 13.1 | 29.6 | 11.8 | 17.8 | 3.1 | 0 | 664 | Sauteing |
| | 5 | 13.8 | 13.1 | 4.9 | 8.2 | 6.6 | 4.1 | 919 | Sauteing |
| | 6 | 12.8 | 35.8 | 14.3 | 21.5 | 7.2 | 3.8 | 718 | Sauteing |
| | 7 | 7.4 | 23.3 | 8.3 | 15.0 | 6.5 | 1.4 | 679 | Sauteing |
| Chicken | 1 | 12.7 | 12.3 | 4.3 | 8.0 | 16.3 | 9.8 | 667 | Roasting |
| | 2 | 13.5 | 25.5 | 7.7 | 17.8 | 2.7 | 1.2 | 745 | Deep frying |
| | 3 | 23.2 | 1.8 | 0 | 1.8 | 0 | 0 | 375 | Boiling |
| | 4 | 16.7 | 18.4 | 1.8 | 16.6 | 15.8 | 0.1 | 622 | Deep frying |
| | 5 | 18.1 | 10.6 | 1.7 | 8.9 | 0.3 | 0 | 800 | Roasting |
| | 6 | 15.8 | 9.0 | 2.2 | 6.8 | 9.2 | 7.2 | 810 | Roasting |
| Spam | 1 | 15 | 25.0 | 10.0 | 15 | 1.5 | 0 | 1200 | Steaming |
| | 2 | 14 | 17.0 | 6.0 | 11.0 | 6 | 0 | 850 | Steaming |
| | 3 | 13 | 28.6 | 10.7 | 17.9 | 2.3 | 1.3 | 1000 | Steaming |
| | 4 | 13 | 21.0 | 10 | 11.0 | 1 | 1 | 960 | Steaming |
| Snail | 1 | 21.3 | 0.9 | 0 | 0.9 | 6.7 | 0 | 1110 | Boiling |
| | 2 | 16.2 | 2.2 | 0.2 | 2.0 | 10.5 | 7 | 680 | Boiling |
| | 3 | 12.4 | 0.7 | 0.1 | 0.6 | 10.2 | 3.8 | 788 | Boiling |
| | 4 | 7.5 | 0 | 0 | 0 | 11.2 | 8.7 | 733 | Boiling |
| | 5 | 7.5 | 0 | 0 | 0 | 11.2 | 8.7 | 733 | Boiling |
| | 6 | 16 | 2 | 0 | 2 | 10.5 | 25 | 680 | Boiling |
| Saury | 1 | 11.6 | 21.4 | 6.1 | 15.3 | 5.9 | 4.1 | 290 | Braising |
| | 2 | 14.7 | 15.4 | 3.4 | 12.0 | 3.8 | 3.8 | 436 | Braising |
| | 3 | 13.9 | 17.0 | 2.8 | 14.2 | 10.8 | 10.6 | 524 | Roasting |
| | 4 | 16.4 | 12.5 | 4.7 | 7.8 | 4.7 | 3.1 | 440 | Roasting |

| | | | | | | | | | |
|---|---|---|---|---|---|---|---|---|---|
| | 5 | 17.6 | 15.5 | 4.7 | 10.8 | 6.3 | 4.4 | 520 | Roasting |
| | 6 | 20.3 | 11.6 | 3.5 | 8.1 | 4.8 | 2.9 | 600 | Roasting |
| Eel | 1 | 18.3 | 9.0 | 1.9 | 7.1 | 15.2 | 8.3 | 587 | Braising |
| | 2 | 19.6 | 10.0 | 2.7 | 7.3 | 8.8 | 8.5 | 542 | Braising |
| | 3 | 23.9 | 12.5 | 3.0 | 9.5 | 9.5 | 9.4 | 544 | Braising |
| | 4 | 23.3 | 10.8 | 2.9 | 7.9 | 7.8 | 7 | 368 | Braising |
| | 5 | 18.6 | 11.8 | 2.4 | 9.4 | 9.4 | 9.4 | 663 | Braising |
| | 6 | 11.8 | 6.2 | 0 | 6.2 | 14.5 | 9.2 | 688 | Roasting |
| | 7 | 15.9 | 18.8 | 3.6 | 15.2 | 13.9 | 12.4 | 783 | Braising |
| Mackerel | 1 | 17 | 19.5 | 0 | 19.5 | 7.4 | 7.4 | 668 | Boiling |
| | 2 | 18.1 | 17.9 | 0 | 17.9 | 12 | 12 | 472 | Boiling |
| | 3 | 19.1 | 7.6 | 4 | 3.6 | 5.4 | 5.4 | 315 | Boiling |
| | 4 | 14.7 | 15.2 | 6.1 | 9.1 | 6.7 | 2.7 | 448 | Boiling |
| | 5 | 18.4 | 22.4 | 4.5 | 17.9 | 0 | 0 | 380 | Boiling |
| | 6 | 14.6 | 5.6 | 3.4 | 2.2 | 10.6 | 9.6 | 569 | Boiling |
| | 7 | 13.8 | 20.6 | 6.9 | 13.7 | 7.4 | 1.9 | 394 | Boiling |
| Tuna | 1 | 17.9 | 0.3 | 0.1 | 0.2 | 0.1 | 0 | 337 | Boiling |
| | 2 | 18 | 25.8 | 4.1 | 21.7 | 0.1 | 0 | 394 | Boiling |
| | 3 | 13.6 | 27.4 | 8.3 | 19.1 | 0 | 0 | 182.9 | Boiling |
| | 4 | 17.6 | 14.4 | 4.2 | 10.2 | 0.4 | 0.3 | 401.4 | Boiling |
| | 5 | 15.2 | 2.9 | 1.4 | 1.5 | 2.2 | 0 | 955 | Boiling |
| | 6 | 16.3 | 9.4 | 1.6 | 7.8 | 0.6 | 0 | 376 | Boiling |
| | 7 | 21 | 19.7 | 3.9 | 15.8 | 0 | 0 | 412 | Boiling |
| | 8 | 15.1 | 15.7 | 2.7 | 13.0 | 0 | 0 | 454 | Boiling |
| | 9 | 18.9 | 18.0 | 7.3 | 10.7 | 0.7 | 0.4 | 358 | Boiling |
| Sardine | 1 | 20.7 | 10.1 | 6.8 | 3.3 | 7.6 | 1.5 | 480 | Boiling |
| | 2 | 18 | 13.0 | 3.2 | 9.8 | 0 | 0 | 590 | Boiling |
| | 3 | 20.8 | 17.2 | 3.5 | 13.7 | 0.8 | 0 | 266.7 | Boiling |
| | 4 | 14.3 | 36.4 | 6.4 | 30.0 | 0 | 0 | 629 | Boiling |
| | 5 | 14.3 | 36.4 | 6.4 | 30.0 | 0 | 0 | 629 | Boiling |
| | 6 | 12.9 | 24.0 | 8.0 | 16.0 | 7.9 | 4 | 394 | Boiling |

| | | | | | | | | | |
|---|---|---|---|---|---|---|---|---|---|
| | 7 | 17 | 32.0 | 9.6 | 22.4 | 0 | 0 | 472 | Boiling |
| | 8 | 20.2 | 8.3 | 2.5 | 5.8 | 7.2 | 3.5 | 590 | Roasting |
| | 9 | 15.9 | 8.9 | 2.7 | 6.2 | 8.7 | 6.1 | 640 | Boiling |
| Clam | 1 | 15.5 | 2.2 | 0.5 | 1.7 | 11.1 | 3 | 931 | Roasting |
| | 2 | 11.6 | 1.1 | 0.4 | 0.7 | 3.4 | 0 | 487 | Boiling |
| | 3 | 14.2 | 22.3 | 2.8 | 19.5 | 4.1 | 0 | 680 | Boiling |
